# Low-cost LiDAR scanning data for geometric and volume analysis of 3D-printed concrete-plastic elements

**DOI:** 10.1016/j.dib.2025.111799

**Published:** 2025-06-17

**Authors:** Piotr Kędziorski, Aneta Skoratko, Jacek Katzer, Paweł Tysiąc, Marcin Jagoda, Machi Zawidzki

**Affiliations:** a Faculty of Civil Engineering, Environmental and Geodetic Sciences, Koszalin University of Technology, Śniadeckich 2, 75-453 Koszalin, Poland; b Faculty of Geoengineering, University of Warmia and Mazury in Olsztyn, Prawocheńskiego 15, 10-720 Olsztyn, Poland; c Faculty of Civil and Environmental Engineering, Gdańsk University of Technology, Gabriela Narutowicza 11/12, 80-233 Gdańsk, Poland; d Institute of Fundamental Technological Research, Polish Academy of Sciences, Pawińskiego 5B, 02-106 Warsaw, Poland

**Keywords:** Low-cost LiDAR, 3D printing, Point cloud analysis, Fractals, Concrete

## Abstract

This dataset presents experimental data on the use of low-cost LiDAR scanners (integrated with iPads and iPhones) to evaluate the deformation of plastic-concrete specimens with fractal-based cross-sections. The specimens were created using 3D printed lost formwork and concrete. The dataset includes mesh models acquired using low-cost LiDAR technology and photogrammetry before and after the loading tests. This allows for the evaluation of geometric deformations and volume changes in specimens of varying cross-sectional complexity.

The measurements were performed in a controlled laboratory environment, where LiDAR-based volume calculations were compared with high-precision photogrammetric reference data. The dataset includes information on scanning conditions, point cloud processing techniques, and measurement errors, providing insight into the accuracy and repeatability of low-cost LiDAR technology in structural assessment.

This dataset is valuable to researchers investigating low-cost metrology, LiDAR-based strain monitoring, and the application of consumer-grade scanning technology in civil engineering and materials science. It enables further analysis of the accuracy of mobile LiDAR for measuring complex geometries and deformations of structures.

Specifications Table

**Table:** 

Subject	Engineering & Materials science
Specific subject area	Application of low-cost LiDAR in civil engineering metrology: Accuracy and limitations in measuring concrete-plastic columns with fractal based cross-sections
Type of data	Table (*.xlsx format), 3D mesh models (*.stl format)
Data collection	Concrete-plastic specimens with fractal-based cross-sections in 12 different designs were measured. Data acquisition was performed using a LiDAR scanner integrated with an Apple iPad Pro and the Scaniverse app, as well as close-range photogrammetry using a Fujifilm X-S10 camera and Agisoft Metashape software. Measurements were conducted before and after compressive loading to assess deformation. The raw mesh data were processed and cleaned using CloudCompare and Blender software to remove noises.
Data source location	Institution: Koszalin University of Technology City/Town/Region: Koszalin Country: Poland Latitude and longitude (GPS coordinates) for collected data: latitude: 54.204201, longitude: 16.197807
Data accessibility	Repository name: Mendeley Data Data identification number: 10.17632/t4hs5rdxjm.1 Direct URL to data: https://data.mendeley.com/datasets/t4hs5rdxjm/1
Related research article	P. Kędziorski, A. Skoratko, J. Katzer, P. Tysiąc, M. Jagoda, M. Zawidzki: Harnessing low-cost LiDAR scanners for deformation assessment of 3D-printed concrete-plastic columns with cross-sections based on fractals after critical compressive loading, Measurement 249 (2025) 117015. 10.1016/j.measurement.2025.117015.

## 1 Value of the Data

• The dataset provides information for evaluating the accuracy of low-cost LiDAR scanners in structural measurements. Allows researchers to analyze the accuracy and repeatability of LiDAR scanners mounted on Apple devices when measuring 3D printed concrete-plastic columns. Provides an opportunity to test the capabilities of consumer devices in construction metrology applications.
• The dataset includes LiDAR and photogrammetric measurements taken before and after compressive loading. This allows a comparison of the two to assess the accuracy and limitations of measuring small objects with complex geometries based on fractals.
• The dataset can be used to develop and test post-processing algorithms for mesh reconstruction and object volume estimation, contributing to the development of low-cost measurement strategies.
• With the growing influence of 3D printing in civil engineering, the dataset provides information on the behavior of specimens made using this concrete-plastic technology under compressive loading, which will aid in further research on material properties.

## 2 Background

The increasing use of 3D printing in civil engineering is leading to the more frequent use of structures with complex geometries, such as fractals [1]. Research on such elements, conducted to evaluate their suitability for construction and to determine their strength [[2], [3], [4]], has identified the need to accurately analyse their geometry after critical compressive loading. Traditional measurement methods can have difficulty accurately representing their geometry, especially after the specimen has been destroyed. Terrestrial laser scanning (TLS) is one possible solution, but the cost of these devices is high. The advent of low-cost LiDAR scanners integrated into consumer devices (such as iPads and iPhones) has created a potential alternative [5]. Recent studies have demonstrated promising applications of Apple LiDAR for heritage documentation [6], building interior mapping [7], BIM modelling [8], among others [[9], [10], [11], [12], [13], [14]]. However, the accuracy and repeatability of these scanners in scientific applications remains understudied, which has been a major research concern. Sharing the data acquired during research will allow for a better understanding of the original article due to the specific characteristics of the data obtained. Spatial models cannot be fully presented in scientific articles, making it difficult for readers to interpret them. Being able to access and work with the data will allow potential audiences to perform their own analyses and better understand the methodology and results presented in the original article.

## 3 Data Description

The provided dataset contains processed mesh models in STL format (*.stl). It consists of two main folders:

“Before loading” – containing models of specimens measured before applying compressive load.

“After loading” – containing models of specimens after deformation or destruction due to loading.

Each of these folders contains .xlsx files with the detailed results of the volume comparisons and two subfolders:

“LiDAR” – containing models obtained using a low-cost LiDAR scanner.

“Photogrammetric” – containing models generated via photogrammetry.

**Fig. 1:**
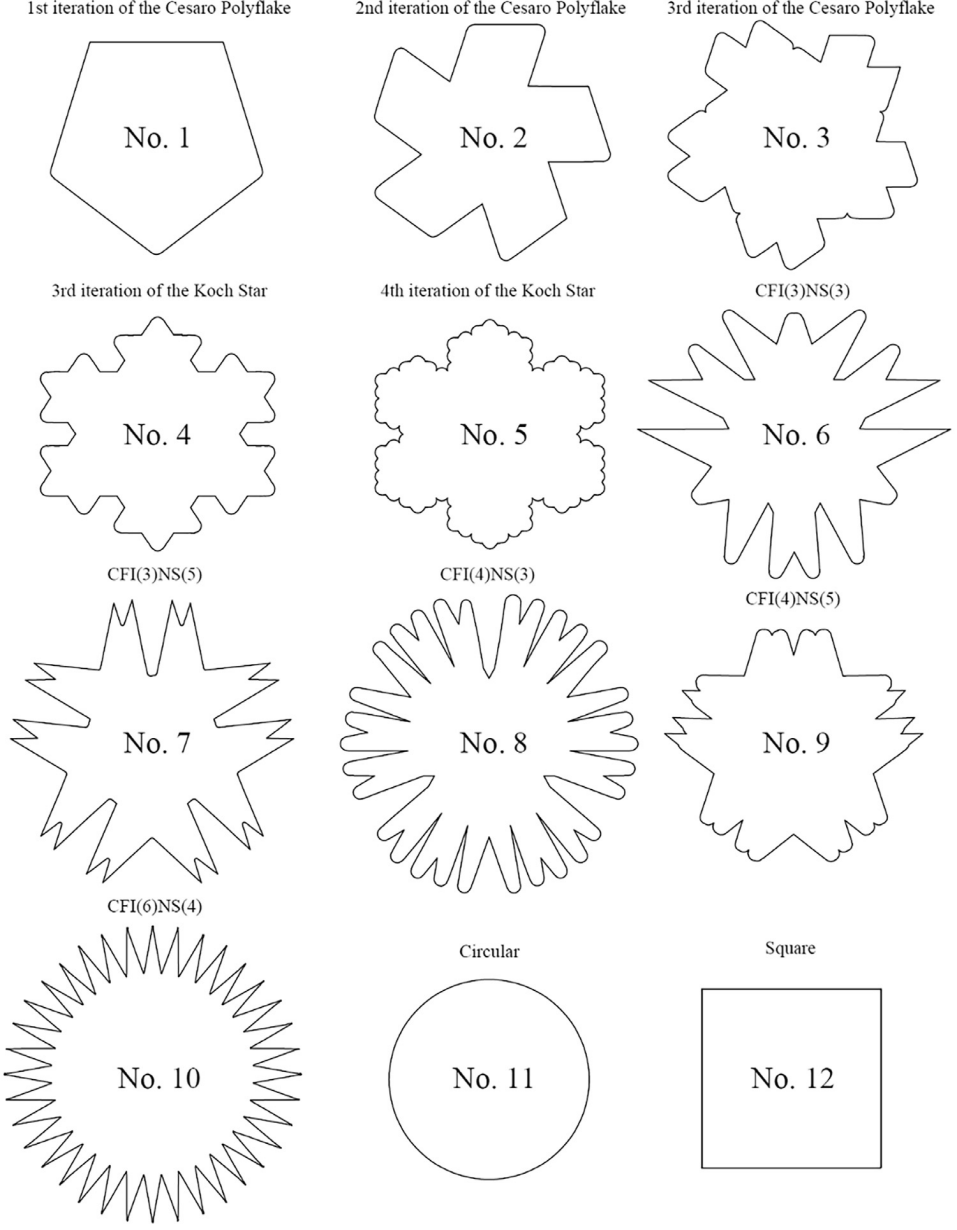
Cross-sections of concrete-plastic columns.

The file naming convention follows the format xx-yy-zz.stl, where: xx – corresponds to the cross-section number of the specimen (as shown in Fig. 1). yy – represents the specimen count number for a given cross-section. zz – indicates the scanning method, either LiDAR or Photogrammetric.

## 4 Experimental Design, Materials and Methods

Prior to the actual data collection, a pilot study was conducted to determine the optimal measurement method that would yield the best results. Prior to the actual data acquisition, a pilot study was conducted to determine the optimal measurement method. Initial tests showed that the ad hoc scanning approach provided unsatisfactory results. Therefore, four critical factors were examined to establish an effective measurement workflow.

**Fig. 2:**
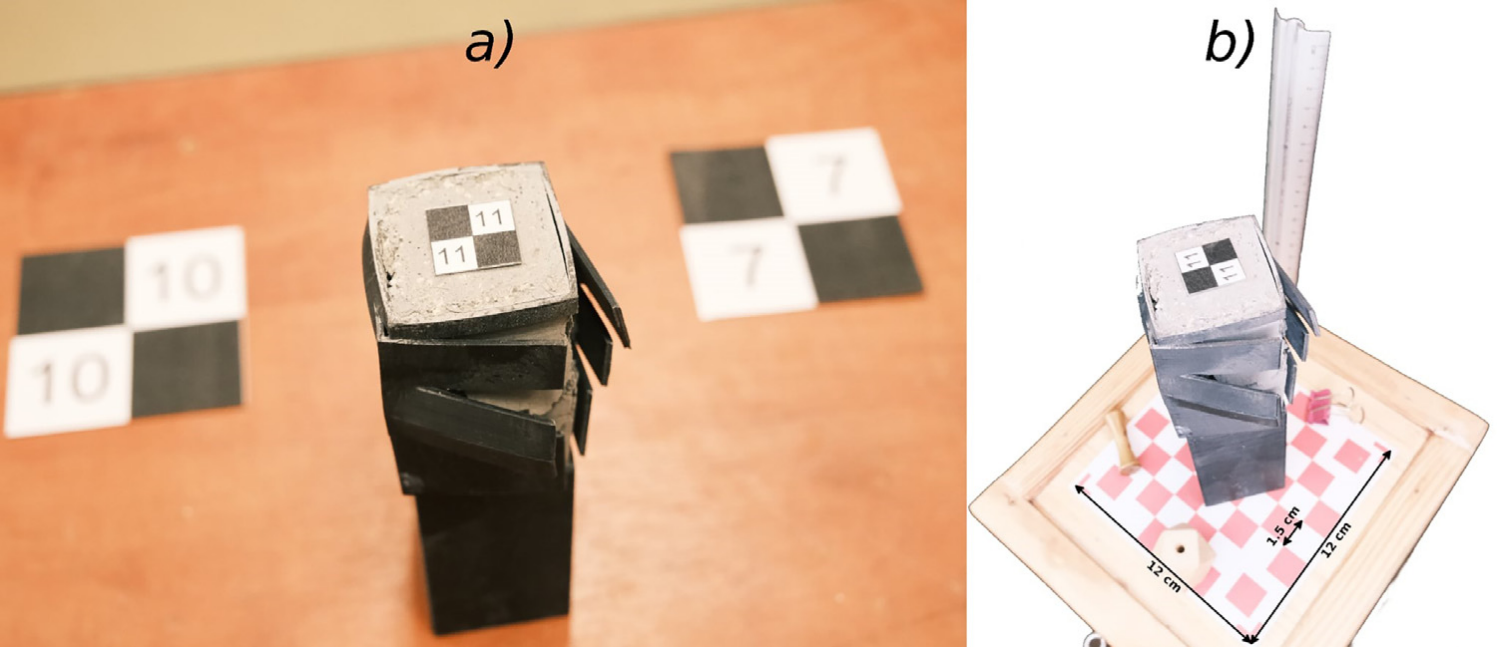
Example photos of the measurement scene. a) In the a hoc approach. b) After implementation of the proposed approach.

First, the number of scanning passes around the specimen was increased from three to four, while placing the specimen on a narrow pedestal, allowing the scanner to access its lower part more effectively. Second, checkerboard markers were added to enhance the photogrammetry based postprocessing in the Scaniverse app. Third, spatial elements were introduced into the measurement scene to collapsing the planarity and improve the performance of the LiDAR sensor. Finally, lighting conditions were improved. The low-cost LiDAR performs poorly in low-light conditions [15]. Strong illumination was used to reduce surface uniformity, enhance visibility of the 3D-printed structure, and reveal surface irregularities such as dirt or texture [16]. The view of the measurement scene before and after introducing the proposed measurement methodology is shown in Fig. 2. The methodology developed in this way was used in both Stage 1 and Stage 2 of the study.

Stage 1 - Assessment of measurement accuracy and repeatability.

Data were collected in two measurement campaigns. The first was to evaluate the accuracy and repeatability of scanning with a low-cost LiDAR scanner and photogrammetric method. For this purpose, 12 concrete samples were made, each with a different shape corresponding to the cross-sections shown in Fig. 1. The samples were created from CAD models, so their geometry and volumes were known. Each sample was measured five times using both methods:

• LiDAR: scanning using Apple iPad Pro (5th gen, 128 GB, iOS 16.6.1) with Scaniverse app.
• Photogrammetry: Fujifilm X-S10 (APS-C, 26.1 MP), 250 images/specimen, burst mode (3 photos per second), f/14, ISO 5000, 1/125 s, 16 mm lens, handheld at approximately 0.2 with a ground pixel size of about 0.047 mm. Image processing in Agisoft Metashape.

**Fig. 3:**
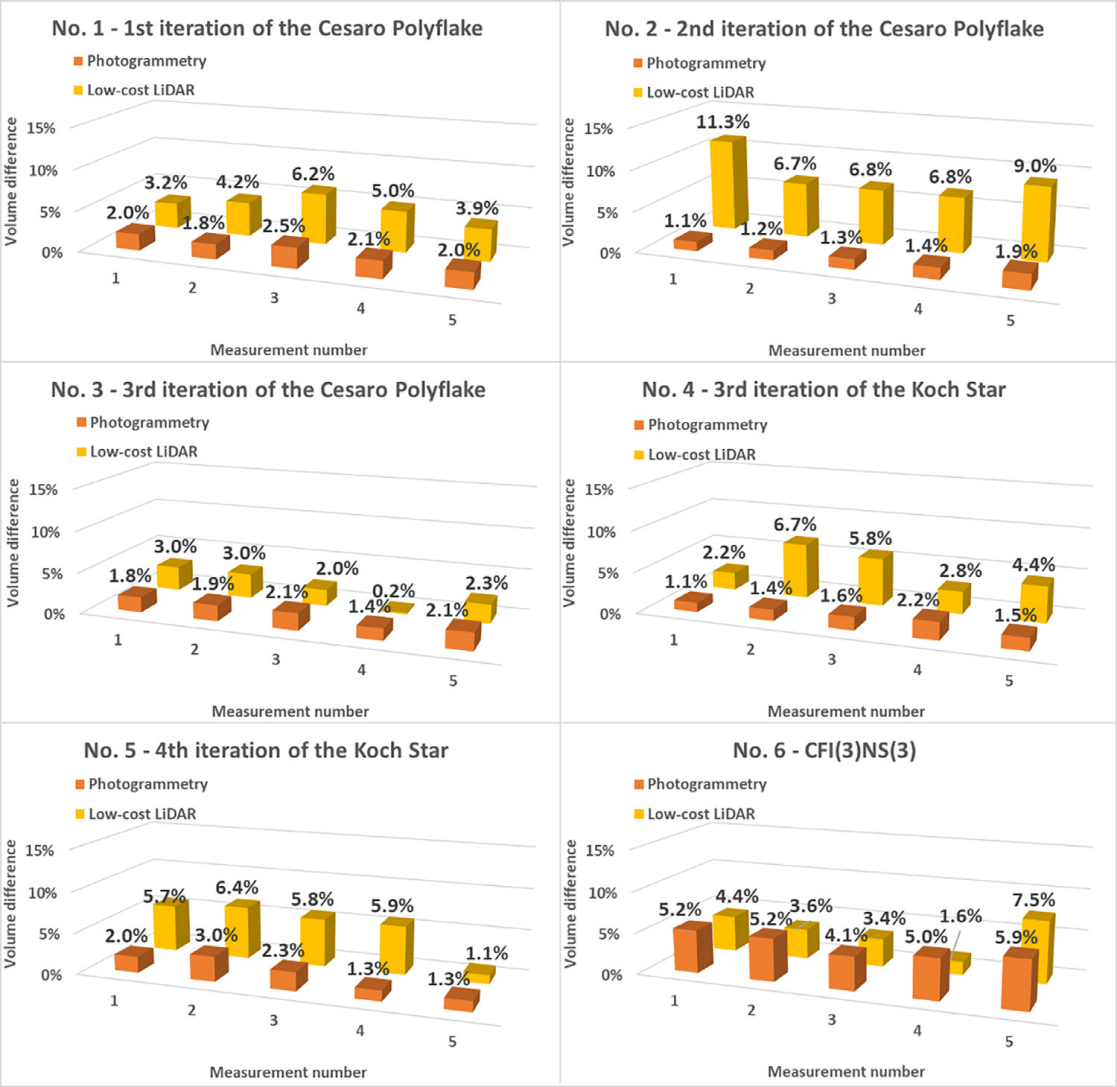
Measurement results of specimens before loading, cross-section No. 1 – 6.

**Fig. 4:**
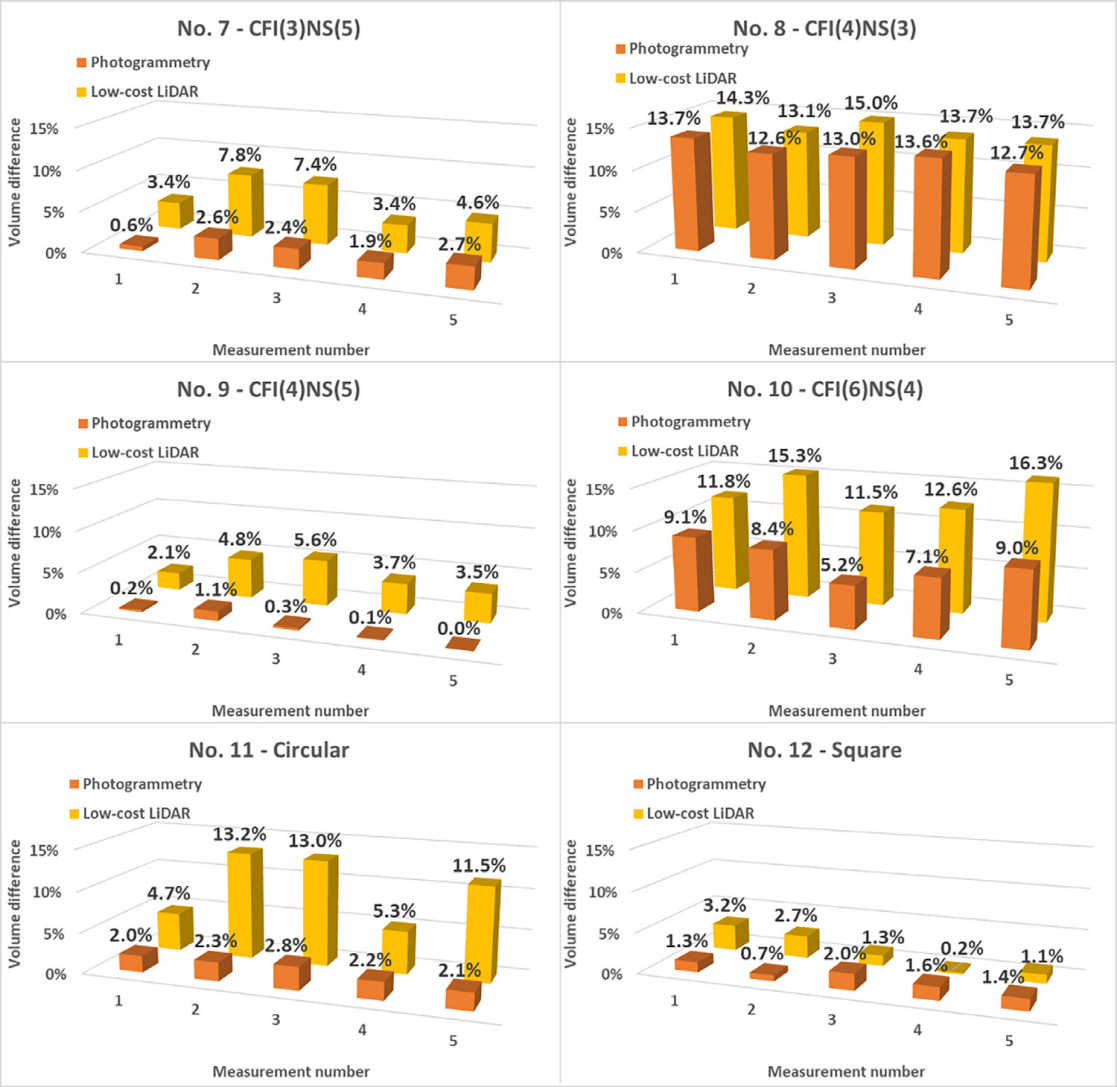
Measurement results of specimens before loading, cross-section No. 7 – 12.

Raw data was cleaned in CloudCompare and Blender to preserve only the sample geometry. Model volumes were then calculated using a Python script in Blender that divided the mesh into tetrahedrons and summed their volumes to determine the total volume of the sample. The results were compared to model volumes (CAD) and between measurement methods. A comparison of the results is shown in Figs. 3 and 4 and detailed results are included in the .xlsx files of the dataset.

Stage 2 - Measurements after loading

In the second stage, the specimens were subjected to load tests after 28 days of concrete curing. A press with a maximum load of 300 kN was used for the test:

• The load was applied axially with respect to the height of the columns.
• An initial load of 100 N was maintained for 30 s to allow the specimen to stabilize.
• The load was then gradually increased at a displacement rate of 0.5 mm/min until the specimen was completely destroyed or reached a displacement of 5.6 mm (3.5% strain).

**Fig. 5:**
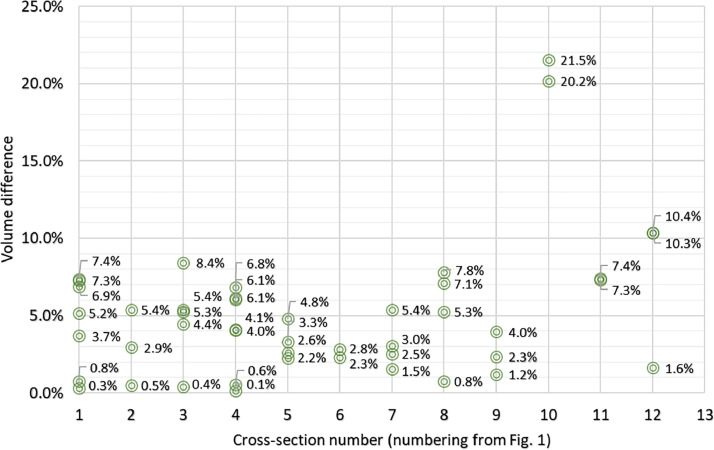
Volume differences depending on the cross-sectional shape of the sample (numbering from Fig. 1).

A total of 46 specimens of different types were scanned after the test, using the same measurement method as before loading. Photogrammetry was chosen as the reference method because it was found to be more accurate in the first stage. The volumes obtained were compared and the results are shown in Fig. 5, detailed results are included in the .xlsx files of the dataset. In addition, the accuracy of the LiDAR mapping of the geometry was evaluated. The distances between the points of the LiDAR and photogrammetric models were calculated in CloudCompare.

## Limitations

None.

## Ethics Statement

The authors have read and follow the ethical requirements for publication in Data in Brief and confirming that the current work does not involve human subjects, animal experiments, or any data collected from social media platforms.

## CRediT Author Statement

**Piotr Kędziorski:** Writing – original draft, Visualization, Validation, Software, Data curation, Conceptualization. **Aneta Skoratko:** Methodology, Investigation, Formal analysis, Data curation, Conceptualization. **Jacek Katzer:** Writing – review & editing, Supervision, Resources, Project administration, Investigation, Formal analysis, Conceptualization. **Paweł Tysiąc:** Writing – review & editing, Validation. **Marcin Jagoda:** Writing – review & editing, Supervision, Project administration, Methodology, Funding acquisition, Formal analysis, Conceptualization. **Machi Zawidzki:** Writing – review & editing, Validation, Formal analysis.

## Data Availability

Mendeley DataDataset for Low-Cost LiDAR Scanning Geometric and Volume Analysis of 3D-Printed Concrete-Plastic Elements (Original data).
